# Predicting potential target genes in molecular biology experiments using machine learning and multifaceted data sources

**DOI:** 10.1016/j.isci.2024.109309

**Published:** 2024-02-23

**Authors:** Kei K. Ito, Yoshimasa Tsuruoka, Daiju Kitagawa

**Affiliations:** 1 Department of Physiological Chemistry, Graduate School of Pharmaceutical Science, The University of Tokyo, Tokyo 113-0033, Japan; 2 Department of Information and Communication Engineering, Graduate School of Information Science and Technology, The University of Tokyo, Tokyo 113-0033, Japan

**Keywords:** Molecular biology, Natural language processing

## Abstract

Experimental analysis of functionally related genes is key to understanding biological phenomena. The selection of genes to study is a crucial and challenging step, as it requires extensive knowledge of the literature and diverse biomedical data resources. Although software tools that predict relationships between genes are available to accelerate this process, they do not directly incorporate experiment information derived from the literature. Here, we develop LEXAS, a target gene suggestion system for molecular biology experiments. LEXAS is based on machine learning models trained with diverse information sources, including 24 million experiment descriptions extracted from full-text articles in PubMed Central by using a deep-learning-based natural language processing model. By integrating the extracted experiment contexts with biomedical data sources, LEXAS suggests potential target genes for upcoming experiments, complementing existing tools like STRING, FunCoup, and GOSemSim. A simple web interface enables biologists to consider newly derived gene information while planning experiments.

## Highlights

• A machine learning system suggests potential target genes for future experiments
• The system was trained on the sequence of experiment descriptions and biomedical data
• Twenty-four million experiments were extracted from PubMed Central’s results sections
• LEXAS is a simple web interface for searching and suggesting biological experiments

## Graphical abstract

**Figure:**
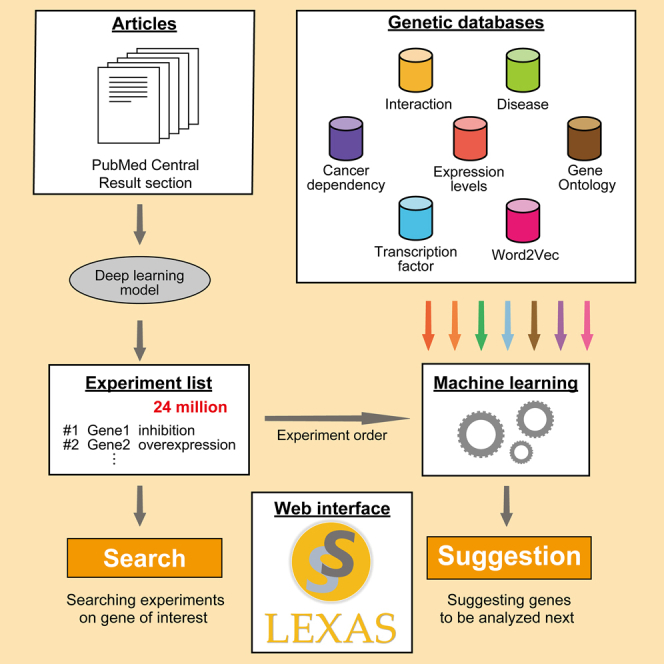

## Introduction

In molecular biology, researchers often face questions like "What gene should we target in the next experiment?" once they have completed an experiment on a gene. To find the answer to this question, they usually consult the relevant literature and biomedical databases to look for potentially functionally related genes. Due to the large amount of literature and databases available today, they spend a lot of time planning their experiments.

There are many text-mining-based applications that can help researchers quickly grasp various gene-related information described in the literature. Some tools display biomedical concepts such as diseases and compounds related to the gene of interest.^1^^,^^2^^,^^3^^,^^4^ Others perform biological event extraction from parts of the literature, such as abstracts, to find the relationships between genes or proteins, such as physical interaction, activation, inhibition, and phosphorylation.^5^^,^^6^^,^^7^^,^^8^^,^^9^^,^^10^ Based on the text-mined information provided by these tools, researchers can hypothesize functionally related genes to analyze in their next experiment.

In addition to displaying text-mined information, many tools have been developed to predict functionally related genes. For example, GeneMania,^11^ FunCoup,^12^ HumanBase,^13^ and HumanNet^14^ predict gene-gene functional relations using information obtained from multiple databases, including protein-protein interactions, expression levels, and co-evolution. GIREM^15^ predicts functionally related genes using only text-mined information from PubMed abstracts. STRING employs both databases and text-mined information to predict functional and physical interactions between proteins.^16^ GOSemSim^17^ calculates the semantic similarity between two genes by applying a graph-based method to the Gene Ontology (GO) hierarchy.^18^^,^^19^ These resources use machine learning models to predict functional associations between genes or proteins, using information in databases as the "gold standard" for training the models. For example, FunCoup uses the protein-protein interaction database iRefIndex^20^ as a gold standard for predicting physical interactions and the signaling pathway database KEGG^21^ for predicting functional interactions in signaling pathways. STRING uses the Complex Portal database^22^ as a gold standard to predict physical interactions. However, none of these existing systems are designed to directly answer the aforementioned question, i.e., they do not suggest the target genes of the next experiment.

Deep reinforcement learning and active learning approaches have already been used to help biologists design experiments. Deep reinforcement learning is used to optimize the parameters of experiments such as nutrient concentrations.^23^ On the other hand, active learning approaches are used to predict genes to be analyzed.^24^ Active learning approaches start by inferring gene networks and then suggesting informative genes for subsequent experiments to improve network accuracy. However, many of these gene networks are constructed based solely on data such as gene expression levels, ignoring prior knowledge from the published literature. Although this allows for broad application across various gene networks or functions regardless of their prior research status, it may compromise the accuracy that could be gained by incorporating knowledge from published literature.^24^ A minority of these approaches, including the robot scientist Adam,^25^ do make use of information from publications, yet their adaptability remains limited. There is still a lack of a comprehensive tool that effectively utilizes information from the literature to suggest genes for future experiments.

**Figure 1:**
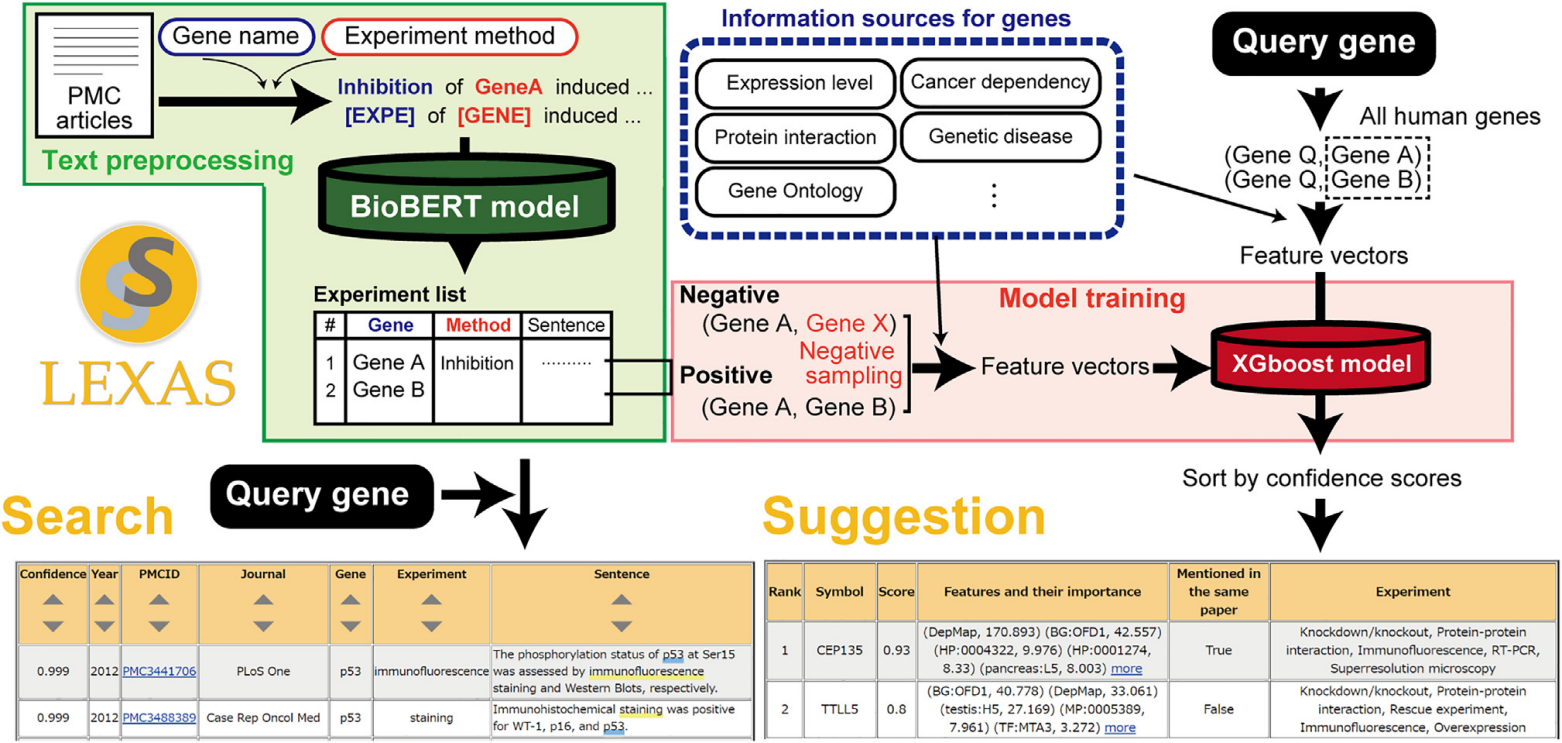
Overview and design of LEXAS Sentences containing at least one gene name and one experiment method were extracted from the result section of articles archived in PubMed Central. The gene name and experiment method were masked by special tokens and then fed into a fine-tuned bio-BERT model for relation extraction. This resulted in the experiment list, which is browsable through the search interface. The context of the experiments was extracted from the experiment list and represented as a tuple. Along with corresponding negative examples, these tuples were used to generate feature vectors for training a prediction model for future experiments on genes. Result tables can be obtained by querying “TP53, immunofluorescence” for the search interface and “Cep63” for the suggestion interface.

In this work, we have developed LEXAS (Life science EXperiment seArch and Suggestion), a system that can suggest genes to be analyzed in the next experiment by using the information on the order of experiments as described in biomedical articles, as well as various biomedical data sources. We first obtained comprehensive gene-related experiment descriptions from the articles archived in PubMed Central using a deep-learning-based natural language processing model. We then focused on the sequential order of experiments in each article and trained machine learning models that can predict the target gene of the next experiment from the target gene of the previous experiment. LEXAS provides results that align well with the researcher’s decision-making process, offering a useful complement to existing tools. LEXAS is available at https://lexas.f.u-tokyo.ac.jp as a web application. The overview of LEXAS is shown in Figure 1.

## Results

### Extraction of experiment information from the literature

**Figure 2:**
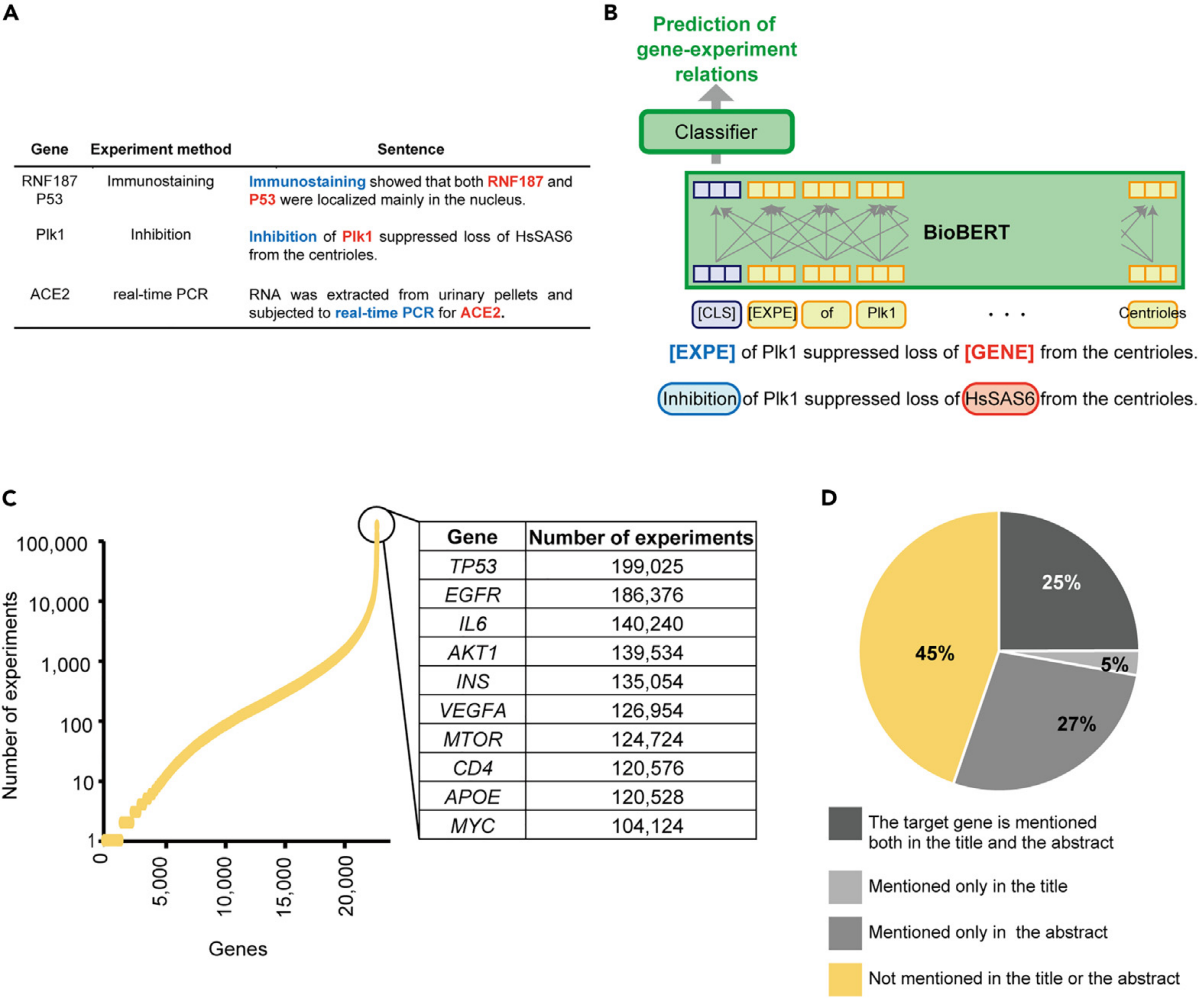
Experiment retrieval from full-text biomedical articles (A) Examples of biological experiments defined as research activities in which genes are analyzed using experiment methods. (B) Extraction of gene-experiment relations by using BioBERT. A sentence in which a gene name and an experiment method are masked with [GENE] and [EXPE] is fed into the fine-tuned BioBERT model to predict whether the gene and the method are in a gene-experiment relation. (C) Cumulative scatterplot indicating the number of experiments in which a gene was analyzed. The top 10 genes with the highest number of experiments are shown in the table. (D) Pie chart depicting the percentage of target genes mentioned in the titles or the abstracts. See also Figure S1 and Tables S1, S2, and S3.

We first extracted information on gene-related experiments from the biomedical literature. In this study, we define an experiment as a research activity in which genes or proteins are analyzed using a certain experiment method (Figure 2A). For example, the sentence "Immunostaining showed that both RNF187 and P53 were localized mainly in the nucleus" indicates that "immunostaining" was performed on “RNF187” and "P53."

Note that not every combination of gene names and experiment methods mentioned in the same sentence indicates that the experiment has been performed on the gene. For example, the sentence "Inhibition of Plk1 suppressed loss of HsSAS6 from the centrioles" contains one experiment method ("inhibition") and two gene names ("Plk1", "HsSAS6"). However, this sentence describes only one experiment in which inhibition was applied to Plk1, not to HsSAS6. Thus, treating all combinations of gene names and experiment methods as mentions of experiments leads to many false detections of experiments. To predict whether or not a pair of a gene name and an experiment method is indeed in a *gene-experiment* relation, we formulated this task as a relation extraction problem and fine-tuned a BioBERT model,^26^ which is a variant of BERT^27^ pretrained with PubMed articles (Figure 2B).

To fine-tune the BioBERT model, we created a gene-experiment relationship dataset. This dataset consisted of randomly sampled 1,600 pairs of a gene and an experiment method mentioned in the same sentence. Each pair was annotated with a label indicating whether the experiment method was performed to analyze the gene. Of the 1,600 pairs, 587 were annotated as positive and 1,013 as negative, with a Cohen’s kappa coefficient of 0.901. To evaluate the performance of our relation extraction model, we calculated the F1 score using 5-fold cross-validation. We increased the number of annotations in increments of 100, from 100 to 1,600 pairs of genes and experiments. Diminishing returns in performance were observed before 1,600 annotations (Figure S1), indicating that additional annotations would not significantly improve the performance. At this point, the precision of the relation extraction was 0.824, with a recall of 0.810. As a baseline, a precision of 0.271 and a recall of 1.0 can be obtained by considering every combination of a gene name and a method in each sentence to indicate the gene-experiment relation. Note that gene-experiment relations can be described not within a single sentence but across multiple sentences. We leave such relations for future work, and the recall reported here is only an upper bound.

We applied this model to all sentences from the result sections of PubMed Central articles that included at least one human gene name and one experiment method (Tables S1 and S2). In total, 24,635,147 gene-method pairs representing gene-method relations were obtained. Hereafter, the extracted gene-method pairs are simply referred to as “experiments.”

In our experiment collections, 24,226 genes were targeted at least once in the experiments, and 19,008, 12,818, 4,035, and 435 genes were targeted more than or equal to 10, 100, 1,000, and 10,000 times, respectively (Figure 2C). The top 10 target genes with the highest number of experiments were *TP53*, *EGFR*, *IL6*, *AKT1, INS*, *VEGFA*, *MTOR, CD4*, *APOE*, and *MYC* (Figure 2C; Table S3).

We also found that only about 55% of the target genes were mentioned in the title or the abstract (Figure 2D). The remaining 45% of the target genes were only mentioned in the main text.

### Collecting consecutive experiment pairs for training machine learning models

Our goal is to develop a machine learning model that can recommend genes for analysis following an experiment on a given gene. A gene rarely works alone in a cell but more often works with other genes as a functional module.^28^ Therefore, many molecular biologists conduct research that focuses not on a single gene alone but with potentially related genes.^28^ For example, if a researcher focuses on gene A, he/she may next study gene B, which he/she thinks is part of the same functional module as gene A. The selection of gene B is based on various factors, such as the known interaction of protein B with protein A, its common association with the same congenital disorder as gene A, or its similar patterns of tissue expression patterns. By training a machine learning model to predict the next target gene (from gene A to gene B), the model can mimic the researchers' decision-making process in selecting genes for analysis.

This idea is based on the hypothesis that the order of the experiment descriptions reflects the actual order in which the authors conducted the experiments. To test this hypothesis, we randomly selected 300 pairs of consecutive experiment descriptions, each from a different research article. We then manually reviewed the text of the articles to check if the experiments were indeed performed sequentially.

Of the 300 pairs, 167 described experiments on the same gene, whereas 133 pairs described experiments on different genes. For the 167 same-gene pairs, 108 (64.7%, 95% confidence interval [CI]: 57.5%–71.9%) described sequentially performed experiments. However, the remaining 59 same-gene pairs did not refer to sequentially performed experiments—49 of them referred to the same experiment (for instance, the consecutive sentences "We depleted gene X using siRNA" and "Depletion of gene X caused … "). In contrast, for the 133 pairs describing different genes, 122 (91.7%, 95% CI: 86.5%–96.2%) showed a sequential relationship between the experiments. Therefore, we conclude that for descriptions associated with different genes, there is a significant match between their sequence and the actual order of experiments. In training our machine learning models, we exclusively utilized the pairs describing different genes.

**Figure 3:**
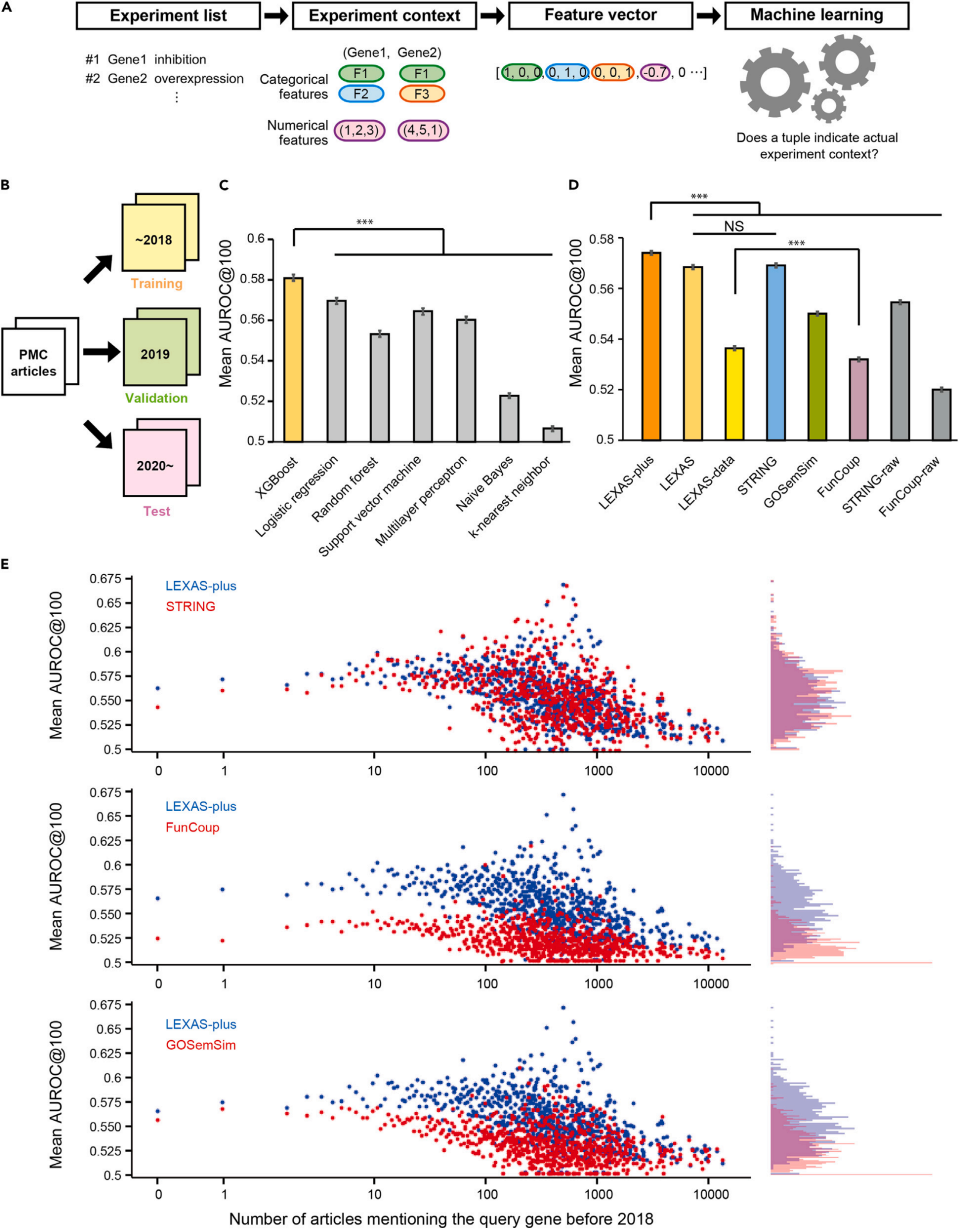
Training and evaluation of machine learning models (A) Schematic illustration of the flow for training a machine learning model using the experiment information extracted from articles. Tuples indicating the context of the experiments were generated from the experiment list and converted into feature vectors. These feature vectors were then used to train the machine learning models. (B) Schematic illustration of data division. Experiments extracted from PMC articles published up to 2018 were used as the training dataset, whereas experiments from 2019 articles were used for validation, and experiments from articles after 2020 were used to test several related tools. (C) Comparison of prediction accuracy between algorithms. The area under the ROC curve (AUROC) @100 was calculated using seven different models. Data are presented as the mean AUROC@100 ± 95% confidence interval (n = 8,278). (D) Comparison of prediction accuracy between our models and other resources. The area under the ROC curve (AUROC) @100 was calculated using eight different models. Data are presented as the mean AUROC@100 ± 95% confidence interval (n = 13,381). (E) Scatterplots depicting the mean AUROC@100 according to the number of articles mentioning the query gene before 2018. Mann-Whitney tests with Bonferroni correction were used in (C) and (D) to compare the mean and obtain the p value. ∗∗∗p < 0.001; NS, p > 0.05. See also Figure S2 and Tables S4 and S5.

Each pair of two consecutive experiments is represented as a tuple consisting of two elements, where the first element is the target gene of an experiment, and the second element is the target gene of the following experiment (Figure 3A). The tuples representing the experiments described in the articles up to 2018 were used to train the machine learning models (628,965 tuples). The tuples representing the experiments described in 2019 were used for validation (63,850 tuples) and those described between 2020 and 2023 (221,318 tuples) were used for evaluation (Figure 3B).

### Constructing feature vectors

To reduce the computational resources for training the model, we transformed the task of predicting the gene to be analyzed from a set of human genes into a set of binary classification problems by using a negative sampling approach.^29^ For each tuple representing experiment contexts, three negative examples were generated by random sampling. We trained machine learning models to classify whether or not a tuple indicated actually described consecutive experiments.

Feature vectors were constructed using the data sources listed in Table 1. The data sources included categorical features such as those from the Gene Ontology,^30^ genetic diseases,^31^ and protein-protein interactions,^32^ as well as numerical features such as expression levels^33^ and gene dependency in cancer cells.^34^ The selection of these data sources was based on their frequent use by professional researchers in our institute, their diverse types of information, and their robust data collection methodologies. Each value in the feature vector reflects a relationship between two genes in a tuple. For example, a value corresponding to a categorical feature indicates whether the two genes in a tuple share the feature. A value corresponding to a numerical feature reflects the degree to which the features of the two genes are correlated.

**Table 1:** Information sources used to train machine learning models

Gene feature	Information source	Type	Dimension	Used to train LEXAS-data
Chromosome location	HGNC^35^	Categorical	46	Yes
Phenotypes of knockout mice	Mouse Genome Informatics^36^	Categorical	4165	Yes
Subcellular localization	Human Protein Atlas^37^	Categorical	58	Yes
Protein-protein interaction	iRefIndex^20^	Categorical	14131	Yes
Transcription factor	ENCODE^38^	Categorical	180	Yes
Phenotypic abnormalities in human	Human Phenotype Ontology^31^	Categorical	3257	
Genetic diseases	Online Mendelian Inheritance in Man, Orphanet	Categorical	83	
Results of sparse matrix learning for the DepMap data	Webster^39^	Categorical	218	
Biological process, molecular function, and cellular component	Gene Ontology^19^	Categorical	4313	
Cancer cell growth under CRISPR/Cas9 mediated suppression of genes	DepMap^34^	Numerical		Yes
Expression levels among cancer cell lines	DepMap	Numerical		Yes
Expression levels among tissues	Human Protein Atlas	Numerical		Yes
Similarity of the usage of gene terms in the MEDLINE abstracts	Word2Vec	Numerical		

This table summarizes the diverse gene features incorporated into the LEXAS model, detailing their sources, types, dimensions, and whether they were used to train the LEXAS-data model.

### Evaluation of the model performance

We evaluated the performance of our models by predicting what gene should be examined next for each query gene and calculating the mean of the area under the ROC curve (AUROC). In the validation and test processes, a suggested gene was considered correct if it was indeed analyzed just after the query gene in the validation and test datasets, respectively. It should be noted that the AUROC scores computed in this evaluation are only approximations of the true accuracy of gene suggestion, because the absence of the suggested gene in the validation/test set does not necessarily mean that the gene is not a reasonable target in the next experiment. A more direct evaluation may be possible by having molecular biology researchers manually inspect the results, but such an evaluation will be small scale compared with the present evaluation. Here, we employed an approximate but large-scale approach for evaluation.

For the validation stage, we first trained several machine learning algorithms using the experiments described up to 2018 and evaluated the results using the experiments described in 2019. The algorithms used were XGBoost, logistic regression, support vector machine, random forest, multilayer perceptron, Naive Bayes, and k-nearest neighbor. Among these algorithms, logistic regression had the highest AUROC, followed by XGBoost (Figure S2A).

We also calculated AUROC@1000 and AUROC@100 to assess whether the true positive gene appeared in the top 1,000 or top 100 suggested genes. These metrics are important because they represent how often the correct gene is among the top suggestions, which is crucial for a recommendation system.^40^ Our results showed that XGBoost has significantly outperformed the other algorithms in the metrics (p < 0.001, n = 8278) (Figures 3C and S2B; Table S4). For comparison, we also implemented a baseline model that simply ranks genes based on the number of experiments in the literature published up to 2018. This baseline model scored lower than XGBoost in terms of AUROC@100 (mean difference: 0.044, 95% CI: 0.042–0.046). We, therefore, chose XGBoost as our final model. Hereafter, we use AUROC@100 to evaluate our model and other resources.

For the test stage, we then compared the XGBoost model, which is named LEXAS, with other popular resources using the test dataset. Among many tools that predict functionally related genes, we chose GOSemSim, STRING, and FunCoup for comparison because they can predict the functional relationship between genes in general, not specific to a disease or a tissue. In addition, we applied the Random Walk with Restart (RWR) method,^41^ a network-based method that calculates node-to-node proximity within a network, to STRING and FunCoup networks. The application of RWR allows us to assess the degree of relatedness or similarity between genes more comprehensively within complex networks. The original STRING and FunCoup data are denoted as “STRING-raw” and “FunCoup-raw,” respectively.

We compared the mean of the AUROC@100 score of LEXAS with those of other tools using the test data. We found that the AUROC score of LEXAS (0.568) was significantly higher than those of STRING-raw (mean difference: 0.013, 95% CI: 0.009–0.017), FunCoup (0.038, 95% CI: 0.036–0.041), FunCoup-raw (0.048, 95% CI: 0.045–0.052), and GOSemSim (0.018, 95% CI: 0.016–0.021) (p < 0.001, n = 13381, Mann-Whitney U test) (Figures 3C; Table S5). However, the AUROC score of LEXAS and STRING (−0.001, 95% CI: −0.004–0.002) did not show a significant difference.

We next attempted to improve the LEXAS model by introducing the information from STRING, FunCoup, and GOSemSim. This new model, LEXAS-plus, showed significantly higher performance (0.573) compared with the other models including STRING (mean difference: 0.005, 95% CI: 0.002–0.008) (p < 0.001). In addition, we developed a model called LEXAS-data, which only uses information from relatively objective databases and FunCoup, where all data are derived from comprehensive analyses such as RNA sequencing or mass spectrometry. The AUROC@100 score of LEXAS-data (0.536) was higher than that of FunCoup alone (mean difference: 0.006, 95% CI: 0.004–0.008, p < 0.001) (Figure 3D).

In the aforementioned evaluation, the gene that was examined just next to the query gene was considered a true positive. However, it is important to test whether the models can predict not only the next but also all the following genes. To test this, we considered all the following genes in the article as true positives and recalculated the metrics. In the validation process, XGBoost again showed the best score (Figure S2C; Table S4). In the test process, the AUROC score of LEXAS (0.576) was significantly higher than those of STRING (mean difference: 0.006, 95% CI: 0.004–0.008), STRING-raw (0.019, 95% CI: 0.017–0.021), FunCoup (0.045, 95% CI: 0.044–0.046), FunCoup-raw (0.044, 95% CI: 0.042–0.046), and GOSemSim (0.028, 95% CI: 0.026–0.030) (p < 0.001, n = 13381, Mann-Whitney U test) (Figure S2D; Table S5). The AUROC value of LEXAS-plus (0.581) was the highest among all tested models including LEXAS (mean difference: 0.005, 95% CI: 0.003–0.006) (Figure S2D; Table S5). These results suggest that LEXAS and LEXAS-plus can reliably predict not only the genes examined just next but also those examined in later experiments following the query gene.

### The relationship between suggestion accuracy and the number of descriptions in the past literature

Next, we tested the relationship between gene suggestion accuracy and the number of descriptions in the past literature. We illustrated the relationship between the number of descriptions and the mean AUROC scores as shown in Figure 3E and compared the AUROC scores. We observed a trend where the LEXAS-plus model consistently outperformed the GOSemSim and FunCoup. However, when compared with STRING, no clear trend was evident overall. Nevertheless, for query genes mentioned fewer than 10 times in the literature, LEXAS-plus tends to outperform STRING. These results suggest that LEXAS-plus is a highly effective model for suggesting genes, particularly those with limited mentions in the existing literature.

### Effect of subcellular localization, molecular function, and biological process of gene transcripts on the suggestion accuracy

We also investigated whether the prediction performance was affected by the category of the gene transcripts, such as subcellular localization, molecular function, and biological process. We used Gene Ontology annotations and selected the top 22 terms by the number of genes annotated to each term. We calculated the mean of the AUROC score for genes annotated with each term.

**Figure 4:**
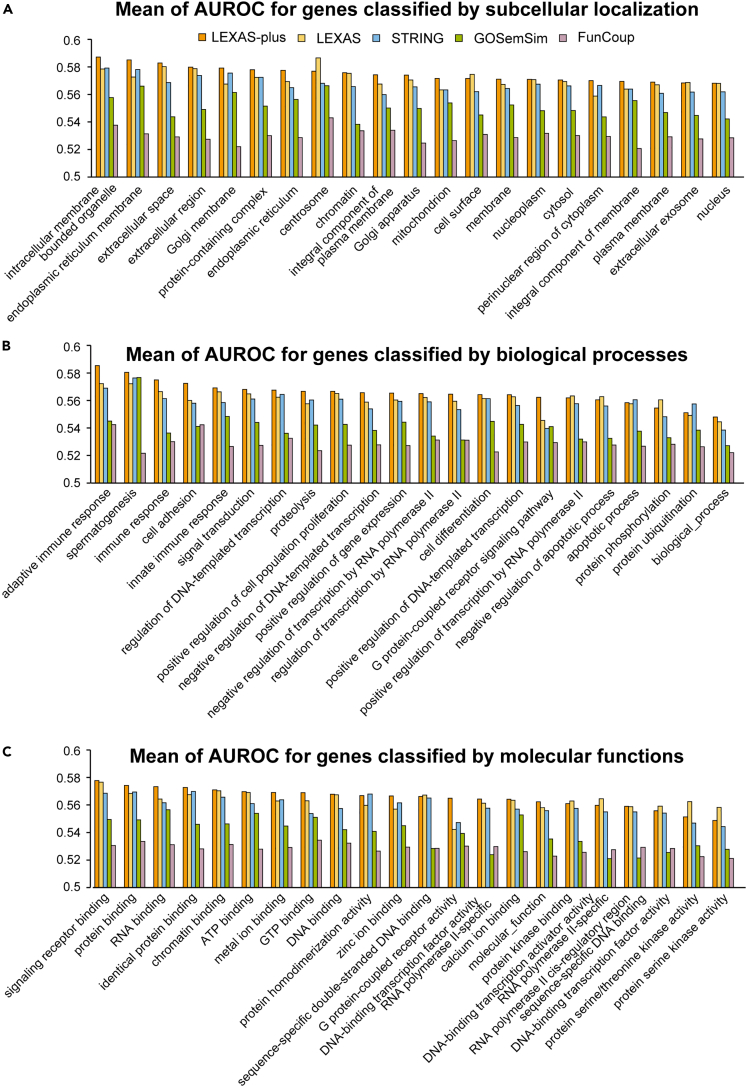
Evaluation of the output of LEXAS system (A) Comparison of prediction accuracy between the categories of query genes classified by subcellular localization. Data are presented as the mean AUROC@100. (B) Comparison of prediction accuracy between the categories of query genes classified by biological process. Data are presented as the mean AUROC@100. (C) Comparison of prediction accuracy between the categories of query genes classified by molecular function. Data are presented as the mean AUROC@100. See also Table S6.

Our analysis showed that for subcellular localization, the predictability was highest for genes localized in the intracellular-membrane-bound organelle and lowest for integral components of the nucleus (Figure 4A; Table S6). LEXAS-plus showed the highest accuracy for genes in almost all subcellular localizations. Regarding biological processes, we found that genes related to the adaptive immune system or spermatogenesis had higher AUROC scores, whereas genes related to protein phosphorylation or ubiquitination had lower scores (Figure 4B; Table S6). Once again, LEXAS-plus showed the highest accuracy for genes involved in most biological processes. In addition, LEXAS-plus also showed the highest AUROC score for genes related to most molecular functions (Figure 4C; Table S6). Therefore, we conclude that the LEXAS-plus model is the optimal model for gene suggestion, regardless of the localization, biological process, and molecular function of gene transcripts.

### Impact of gene features on the suggestion

**Figure 5:**
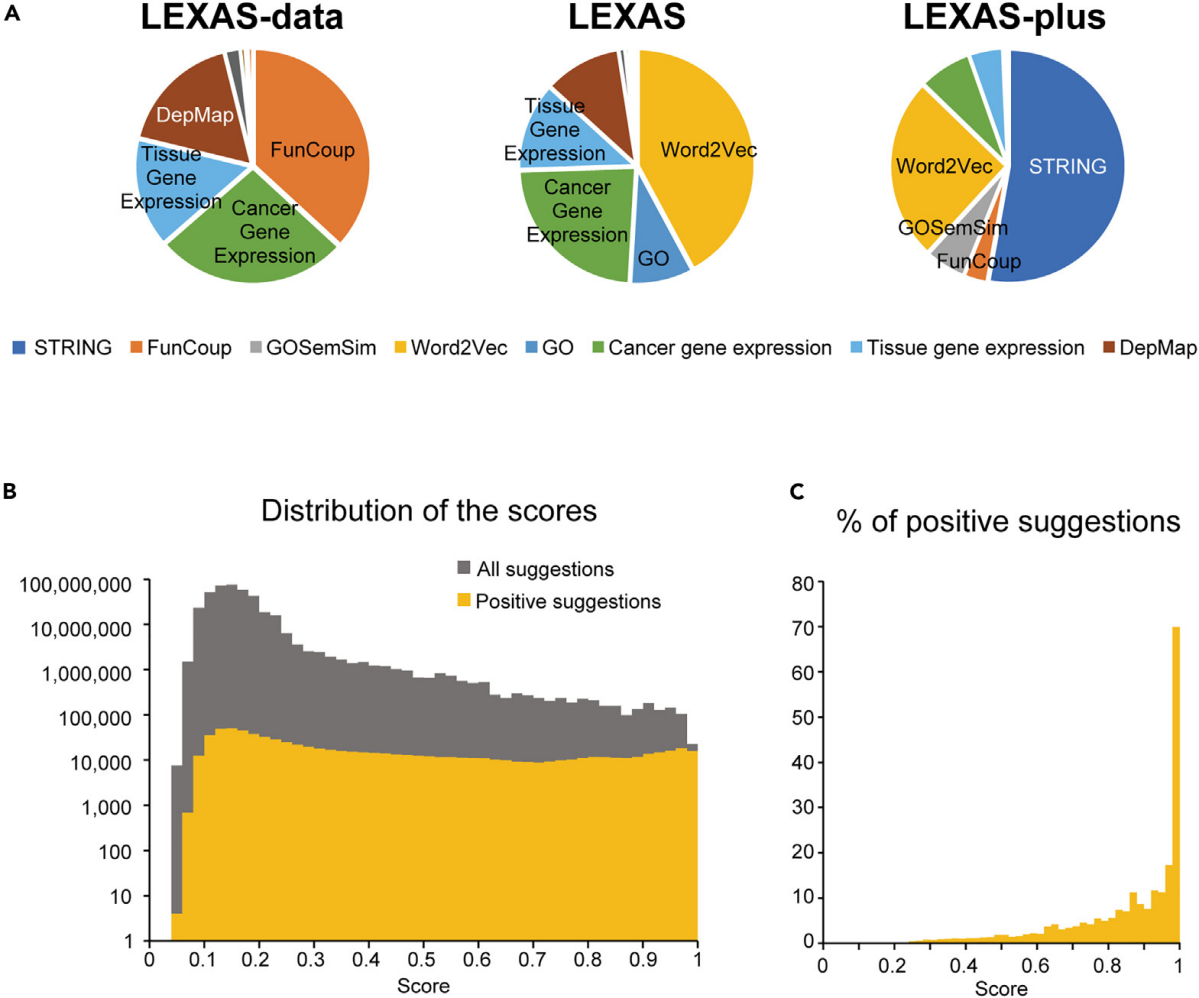
Analysis of important features and scores provided by LEXAS (A) Pie chart depicting the percentage of the used features, i.e., those with the highest SHAP values, to predict the candidate genes to be analyzed in the next experiments by the three models. (B) The distribution of probabilities (scores) for all suggestions and positive suggestions by the LEXAS-plus model. Histogram represents the number of genes with the indicated probabilities. The probabilities were calculated with the LEXAS-plus model trained with the experiments up to 2018. If an experiment on a suggested gene was performed after an experiment on the query gene in any articles, the suggested gene was defined to be true. (C) Histogram represents the ratio of positive suggestions against that of all suggestions with the indicated probabilities. The bin width in the histograms is 0.02 in (B) and (C). See also Figure S3.

To evaluate the impact of gene features on the suggestions, we calculated the SHAP values,^42^ a game-theoretic value that is often used to provide explanations for local outputs of machine learning models such as XGBoost.^43^ For each gene, the SHAP values were calculated for the top 10 suggested genes. Figure 5A shows the frequency of the gene features that have the highest impact on prediction. In the LEXAS-data model trained with objective databases alone, the *pfc* score from the FunCoup database was the most influential, followed by expression levels in cancer and tissue and gene essentiality in cancer cell lines from the DepMap database. In the LEXAS model, on the other hand, the information from Word2vec was the most influential, followed by the expression levels in cancer cell lines. Word2vec is a machine learning approach that represents a word as a vector that reflects its context.^44^ In our model, the Word2vec trained on the full text of PMC articles published up to 2018 was used to vectorize gene names. Therefore, unsurprisingly, the textual context in which the gene name is mentioned is most informative for predicting the genes to be analyzed. In the LEXAS-plus model, the information from STRING was the most influential in about half of the suggestions, and the information sources used to train the LEXAS model were the most influential in the remaining suggestions. These results indicate that the LEXAS models suggest genes to be analyzed by incorporating a variety of gene features.

### Analysis of score distribution in LEXAS-plus model

Figure 5B shows the distribution of probabilities calculated by the LEXAS-plus model. The probabilities for all suggestions were distributed around 0.15, whereas the probabilities for positive suggestions showed a broader distribution. Figure 5C shows the proportion of positive suggestions. Among the suggestions with a probability greater than 0.98, the ratio of positive suggestions is about 70%. On the other hand, the ratio is under 5% among the suggestions with a probability below 0.8. The distribution of probabilities calculated by LEXAS and LEXAS-data models is shown in Figure S3. The probabilities are referred to as “scores” in the user interface.

### A proof of concept for LEXAS

Table 2 shows an example of the output of the LEXAS model trained using the experiments before 2018. In this example, the query was *CEP44*. *CEP44* was identified as a centrosomal gene by proteomics methods in 2011,^45^ but its function was not described until 2020. This table lists the top eight genes with the highest probabilities suggested by our LEXAS model. Interestingly, five out of these eight genes were actually analyzed and demonstrated to be functionally related to *CEP44* in the articles published in 2020^46^ or 2022.^47^ Furthermore, although the other three genes were not analyzed in the articles, the transcripts of *RTTN*, *CEP350*, and *CCDC77* are also localized to the centrosome and appear to be worth analyzing.^48^^,^^49^^,^^50^ These results illustrate that our model can generate reasonable suggestions for the query genes even without any functional information.

**Table 2:** An example of the target gene prediction by the LEXAS model

Rank	Gene	Score	Top 5 features with the highest importance
1	CEP120∗	0.984	DepMap GO term: centriole Cancer gene expression GO term: centrosome iRefIndex: TP53BP2
2	RTTN	0.966	DepMap GO term: centriole GO term: centrosome Tissue gene expression Cancer gene expression
3	CEP350	0.966	GO term: centrosome DepMap GO term: centriole Cancer gene expression Tissue gene expression
4	CEP295∗	0.951	DepMap GO term: centrosome GO term: centriole Tissue gene expression Cancer gene expression
5	CCP110∗	0.949	GO term: centrosome DepMap GO term: centriole Cancer gene expression Tissue gene expression
6	CENPJ∗	0.938	GO term: centrosome DepMap GO term: centriole Cancer gene expression iRefIndex: TP53BP2
7	CCDC77	0.935	DepMap Cancer gene expression GO term: centriole Tissue gene expression niRefIndex: KIAA0753
8	CEP135∗	0.929	GO term: centrosome DepMap GO term: centriole Tissue gene expression iRefIndex: TP53BP2

This table shows the potential target genes after an experiment on *CEP44* predicted by LEXAS model using experiments before 2018. Genes shown with a star were reported to be functionally related to *CEP44* in the articles published in 2020^46^ or 2022.^47^

The top five features with the highest SHAP values were shown in the right column. For example, *CCDC77* was suggested based on the information on the cancer dependency (DepMap), cancer gene expression, an annotation for the GO term “centriole,” tissue gene expression, and protein interaction with KIAA0753. As CEP44 is a centrosomal protein, an interaction with KIAA0753, which is also localized in centrosome,^51^ and the GO term “centriole” are reasonable clues for the suggestion.

### User interface

**Figure 6:**
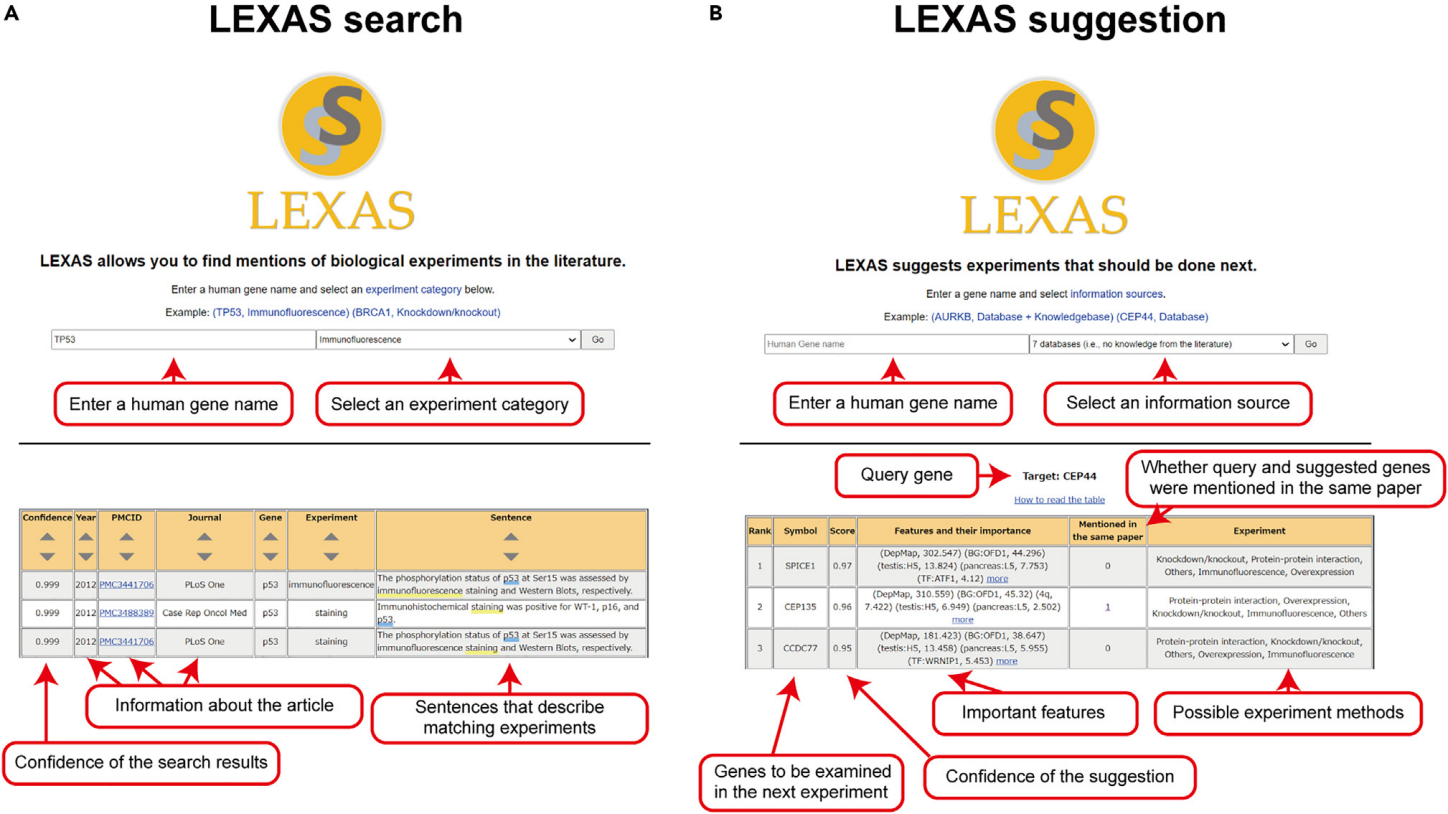
Web interface of LEXAS (A) LEXAS search. The result table is obtained when searching for experiments in which “TP53” was examined with “immunofluorescence.” (B) LEXAS suggestion. The result table is obtained as suggestion for the genes that can be examined after an experiment on CEP44.

Using the collected experiments and trained machine learning models, we built a web application for LEXAS with two interfaces: *search* and *suggestion*. The search interface allows users to retrieve a list of experiment descriptions extracted by the fine-tuned BioBERT model. Given a gene name and the category of an experiment method, the system displays the list of matching experiment descriptions (Figure 6A). The search system offers distinct advantages over previous search methods. First, researchers can quickly access information about experiments on a gene of interest simply by reading a single sentence that describes the experiment, rather than having to read the full text of an article. Second, the system automatically searches for experiment descriptions that include not only the query gene name but also synonymous gene names. Furthermore, we found that 45% of the target genes in experiments were not mentioned in article titles or abstracts (Figure 2D), highlighting the limitations of relying solely on these searches. Our experiment search system can help researchers overcome this limitation by searching the full text. These advantages suggest that our experiment search system is a valuable tool for researchers who want to quickly and accurately identify relevant experiment information for genes of interest.

The suggestion interface allows users to find a list of genes that LEXAS deems should be analyzed after an experiment on a given gene, along with possible experiment methods. The important gene features are displayed along with their SHAP values,^42^ helping the user understand why these genes are suggested (Figure 6B). The system also allows the user to choose between three machine learning models for the suggestion: LEXAS-data, LEXAS, and LEXAS-plus. LEXAS and LEXAS-plus are “plausible” models that use various databases and text-mined information. These models are suitable for those who are seeking plausible suggestions in line with a published body of knowledge. By contrast, the other “exploratory” model (LEXAS-data) was built using the information from objective databases acquired from comprehensive analysis alone. This model is thus suitable for those who are seeking novel and unexpected connections based on relatively objective features.

We will update the data sources and retrain the models at least quarterly in the future to ensure the accuracy and relevance of the search results.

## Discussion

In this work, we developed a gene suggestion system named LEXAS by using machine learning models trained with the information from the experiment descriptions and biomedical data sources. Given a gene of interest, LEXAS produces a list of potentially functionally related genes to be analyzed next. The suggestion accuracy of LEXAS was higher than those of STRING, FunCoup, and GOSemSim.

An important aspect to consider in our study is the potential impact of publication bias on the results provided by our system. Our model relies on the experiment descriptions in the published literature for training. This training dataset inherently carries the publication biases that could affect the performance and generalizability of our model. For example, researchers often report “positive” results more frequently than “negative” ones. This could lead us to miss gene combinations that researchers performed experiments on, but the results did not show the expected outcomes. Furthermore, research on certain genes may be more prevalent in the literature due to historical emphasis or availability of resources, which could also bias our data toward these more popular research areas. We acknowledge this as a limitation of our work.

In this study, we trained machine learning models to predict reasonable target genes in the next experiment. Therefore, in our main evaluation, we considered only the genes analyzed in the immediate next experiment as true positives to demonstrate the effectiveness of our approach (Figure 3). However, it is also possible that the models predict other genes analyzed in the following experiments in the result section of the same article. To test this idea, we employed alternative approach where we regarded all subsequent experiments as true positives rather than just the immediate next experiment (Figure S2). This analysis showed that our LEXAS system is also effective at predicting genes in the rest of following experiments. Thus, although LEXAS was trained to predict just next experiment, it is also useful to predict further experiments as well. In LEXAS web application, a list of potential related genes to be analyzed after the query gene is displayed. Users can select multiple genes from this list to gain insight beyond only the immediate next step.

Our machine learning models predict genes to analyze based on a single query gene. However, this approach could encounter difficulties when dealing with a query gene that possesses multiple functions, because the outputs would include a mix of genes associated with each function of the query gene. An example would be a scenario where a researcher is interested in studying function A of gene X, which is also involved in another function, function B. Querying gene X in our system would return a mix of genes related to either function A or function B. This could obscure the next direction of the study. To mitigate this, the ability to use multiple query genes is beneficial to the gene prediction system. By simultaneously querying gene X with a group of genes related to the function A, we can identify genes that are related to gene X in the context of function A. Extending our LEXAS model to handle multiple query genes would be a promising direction for future research.

The user interface of LEXAS suggestion provides a list of genes as well as reasonable experiments to analyze the suggested gene. To suggest experiment methods, experiment methods were grouped into 20 categories, and a model was built to predict the experiment categories. The LEXAS user interface provides a list of reasonable experiment categories, such as "knockdown" or "immunofluorescence analysis." However, actual experiments are usually more complicated than these categories. Suggesting more specific and concrete experiment methods would be another interesting direction for further study.

### Limitations of the study

This study extracted information of experiments described in single sentences. Consequently, experiments described across multiple sentences were not included in the extraction process. Besides, because LEXAS’s predictions rely on published articles, the results can be influenced by publication biases, which might overrepresent or underrepresent specific areas of biological research.

## STAR★Methods

### Key resources table

**Table:** 

REAGENT or RESOURCE	SOURCE	IDENTIFIER
**Deposited data**		

HGNC (Tweedie et al., 2021)^35^	HUGO Gene Nomenclature Committee	https://www.genenames.org/
Mouse Genome Informatics (Blake et al., 2021)^36^	The Jackson Laboratory	https://www.informatics.jax.org/
Human Protein Atlas (Thul et al., 2017)^37^	The Human Protein Atlas project	https://www.proteinatlas.org/
iRefIndex (Razick, Magklaras and Donaldson, 2008)^20^	VIB Technologies	https://irefindex.vib.be/
Harmonizome^52^	Ma’ayan Laboratory of Computational Systems Biology	https://maayanlab.cloud/Harmonizome/
Human Phenotype Ontology (Köhler et al., 2021)^31^	The Human Phenotype Ontology (HPO) project	https://hpo.jax.org/
Webster (Pan et al., 2022)^39^	Pan et al. (Pan et al., 2022)^51^	https://depmap.org/webster/
Gene Ontology (Carbon et al., 2019)^19^	Gene Ontology Consortium	https://geneontology.org/
DepMap (Meyers et al., 2017)^34^	Broad Institute	https://depmap.org/portal/
Code for the development and evaluation of LEXAS	This paper	https://doi.org/10.5281/zenodo.10115270

**Software and algorithms**		

Python version 3.7	Python Software Foundation	https://www.python.org

### Resource availability

#### Lead contact

Further information and requests for resources should be directed to and will be fulfilled by the lead contact, Kei K Ito (ito-delightfully-kei@g.ecc.u-tokyo.ac.jp).

#### Materials availability

This study did not generate new unique reagents.

#### Data and code availability

• This paper analyzes existing, publicly available data. These accession numbers for the datasets are listed in the key resources table.
• All original code has been deposited at Zenodo and is publicly available as of the date of publication. DOIs are listed in the key resources table.
• Any additional information required to reanalyze the data reported in this paper is available from the lead contact upon request.

### Method details

#### Article retrieval

Full-text articles archived in PubMed Central (PMC) were downloaded in XML format via the PubMed FTP service on January 20, 2023. The “sec” elements (sections) containing the word "result" in their titles were extracted by parsing the articles. The text within these sections was then retrieved for further analysis.

#### Sentence extraction

Sentence segmentation was performed using scispaCy.^53^ Sentences that contained at least one human gene name and one experiment method were extracted using a dictionary-matching algorithm, specifically the Aho–Corasick algorithm, which was implemented as a Python package called "ahocorapy". We used two different term lists for the genes and the experiment methods.

The gene term list consists of 106,953 terms of gene symbols, gene names, alias gene symbols, alias gene names, previous gene symbols, and previous gene names provided by the HUGO Gene Nomenclature Committee (HGNC).^35^ The terms that are less than 3 characters long and 103 stopword-like terms such as “was” and “can” were excluded to avoid false gene detection (Table S1).

The experiment method list consists of 4,303 terms in total, including 3,870 Medical Subject Headings (MeSH) terms and manually compiled 433 terms (Table S2). The manually compiled terms were expanded using a word2vec model trained on the texts of PubMed Central, as described in the 'collection of gene features' subsection in the STAR Methods section. We converted the experiment method terms into vectors and then investigated the top 10 terms most similar to each term. If a term was deemed appropriate and not already included, we added it to the list. The MeSH terms are descendants of the following categories: E05.196 (Chemistry Techniques, Analytical), E05.393 (Genetic Techniques), E05.478 (Immunologic Techniques), E05.200 (Clinical Laboratory Techniques), E05.301 (Electrochemical Techniques), E05.601 (Molecular Probe Techniques), E05.595 (Microscopy), E05.242 (Cytological Techniques), E05.591 (Micromanipulation), E01.370.225 (Clinical Laboratory Techniques), E01.370.350 (Diagnostic Imaging), or E01.370.500 (Mass Screening).

#### Relation extraction for gene and experiment

To train a model for relation extraction between a gene and an experiment method in a sentence, a set of masked sentences was prepared. For each pair of a gene and an experiment method in a sentence, a new sentence was created by masking the gene name and the experiment method with special tokens, [GENE] and [EXPE], respectively. For example, from the sentence, “Inhibition of Plk1 suppressed loss of HsSAS6 from the centrioles.", we replaced the gene name "Plk1" or “HsSAS6” with the [GENE] token and replaced the experiment method "inhibition" with the [EXPE] token. This resulted in the following two sentences:

• [EXPE] of [GENE] suppressed loss of HsSAS6 from the centrioles.
• [EXPE] of Plk1 suppressed loss of [GENE] from the centrioles.

The first sentence is positive, indicating the experiment method [EXPE] was applied to the gene [GENE], while the second sentence is negative, indicating no relationship between the experiment method [EXPE] and the gene [GENE]. In the annotation process, 1,600 masked sentences were randomly chosen and manually annotated by K.K.I whether the experiment method [EXPE] was performed on the gene [GENE] or not. For validating this process, R.Y, a trained PhD student majoring in cell biology, also annotated 100 of these sentences. The agreement between K.K.I and R.Y was assessed using Cohen’s kappa (0.901).

#### Negative sampling

The context of two consecutive gene-related experiments was represented as a tuple that consisted of two genes. Negative examples were generated by replacing the second element of positive examples with a randomly selected gene from all human genes unless the replaced tuple was contained in the positive examples. Three negative examples were generated per one positive example.

#### Collection of gene features

##### Gene locus

The gene locus data was acquired from the HUGO Gene Nomenclature Committee (HGNC).^35^ Information on chromosome number and arm (p or q) was used to generate feature vectors.

##### Gene Ontology

The ontology and annotation data were downloaded from the Gene Ontology. Version 2018-12-01 was used to train models for evaluating performance, while version 2023-01-01 was used to train models for the user interface. The ontology terms annotated on at least 10 human genes were used to generate feature vectors.

##### Mouse genome informatics

Information on genotype-phenotype annotations was downloaded. The mouse gene names were converted to human homologs and then phenotypes annotated on at least 10 human genes were used to generate feature vectors.

##### HPO, OMIM, Orphanet

Information on Human Phenotype Ontology (HPO) annotations version 2023-01-27, which also includes the information on *Online Mendelian Inheritance in Man* and *Orphanet*, was downloaded. The terms annotated on at least 10 human genes were used to generate feature vectors.

##### Human Protein Atlas

Sub-cellular location data based on the results of comprehensive immunofluorescence analysis and gene expression data among 256 tissues obtained from RNA-seq analysis were downloaded from Human Protein Atlas version 22.0. From the RNA-seq data, information on transcripts per million (TPM) was used to generate feature vectors.

##### iRefIndex

Information on protein-protein interactions of human proteins was downloaded from iRefIndex. iRefIndex 19.0 released 2022-08-22 was used to generate feature vectors.

##### DepMap, Webster

Information on expression levels and gene essentiality scores called Chronos among various cancer cell lines were downloaded from DepMap version 22Q4. The gene functions inferred from the DepMap gene essentiality score using a sparse dictionary learning approach called Webster were downloaded from supplementary information of the article.^39^ From the Webster data, the gene-function relationships with loading more than 0.1 or less than -0.1 were used to generate feature vectors.

##### ENCODE

The information about transcription factors and their targeting genes provided by ENCODE was downloaded through Harmonizome.^52^ Each gene was annotated with the information of which transcription factors can affect the expression of the gene.

##### Word2Vec

The word2vec model was trained using the full text of PubMed Central articles. For the validation and test, only articles published up to 2018 were used to train the word2vec model, while all articles were utilized for the web application. The training process utilized the continuous bag-of-words (CBOW) algorithm,^44^ a window size of 10, and a vector size of 100. The word2vec model was implemented using the gensim library in Python.^54^

#### Construction of feature vector

The tuples composed of two gene names were converted into the feature vectors using the information sources including categorical and numerical features listed in Table 1.

For categorical features, three dimensions were assigned for each term in the feature vector. Each of the two genes in a tuple is associated with several feature terms, such as “GO:0006281 (DNA repair)” and “HP:0000252 (microcephaly)”. When the genes in a tuple were both attributed to the feature term, the values corresponding to the term were set to (1, 0, 0). When only the gene in the previous experiment was attributed to the feature, the values were set to (0, 1, 0). When only the gene in the next experiment was attributed to the feature, the values were set to (0, 0, 1). If neither genes were attributed to the feature, the values were set to (0, 0, 0).

One dimension was assigned for each numerical feature in the feature vector. The value indicates the Pearson correlation coefficient between the values of two genes except for word2vec. The value corresponding to word2vec in the feature vector indicates the cosine similarity between the embedding vectors of the two genes in a tuple.

#### Comparison of related tools

Our models and online resources used for the comparison are listed below.

• LEXAS

Our machine learning model trained using the experiments described up to 2018 and GO terms, several gene databases and text-derived information listed in Table 1. For each query gene, all genes were ranked by their probability to be examined after an experiment on the query gene.

• STRING-raw^55^

The STRING database is a comprehensive resource that provides information on protein-protein interactions. It integrates information from numerous sources, including experimental data and text-mined information. The STRING database provides a link score between two genes as "scored links between proteins". STRING v10, updated in 2017, was selected for the comparison so that STRING would not use text-mined information from the articles after 2018. For each query gene, all genes were ranked by scored links between proteins.

• STRING^55^

We applied Random Walk with Restart (RWR) method, a random-walk based method that measures node-to-node proximity in a network,^41^ to the scored links between transcripts in the STRING network. For each query gene, all genes were ranked by their proximities.

• GOSemSim^17^

GOSemSim is a software package that can estimate semantic similarity between gene products based on GO terms. Using GOSemSim, we calculated the semantic similarities between the two genes in a tuple. We chose Wang’s graph-based method^30^ as the calculation method. For each query gene, all genes were ranked by their semantic similarities between the query gene.

• FunCoup-raw^12^

FunCoup provides functional coupling information between two proteins, represented by a probabilistic confidence value called *pfc* (probability of functional coupling). The predictions are based on integrating data from various sources, such as protein-protein interactions and gene co-expression. For each query gene, all genes were ranked by pfc.

• FunCoup^12^

The RWR method was also applied to the pfc of the FunCoup network. For each query gene, all genes were ranked by the proximities.

#### Calculation of the area under the ROC curve

To assess the performance of our machine learning model, we generated 19,393 tuples for each query gene with that gene in the first element and another human gene in the second element. These 19,393 genes were obtained from HGNC, which were annotated as "genes with protein product”. Each tuple was converted into a feature vector and given a probability of representing an actual experimental context using machine learning models. We then ranked the tuples based on their probabilities and calculated the area under the ROC curve (AUROC) for each query gene, using validation or test tuples as true examples. Additionally, we computed AUROC@k, which is similar to the AUROC but only considers the top-k ranked tuples. To calculate this metric, we set the probabilities to 0 for any genes that were ranked below *k*. We also calculated the same metrics for the semantic similarity for STRING and RWR proximity scores for STRING and FunCoup. To break ties, small random values ranging between 0 and 1×10^−^^10^ were added to the score.

In the validation process, genes that were analyzed following the query gene before 2018 but not in 2019 were removed from the result table, because these genes cannot be considered positive or negative examples. Similarly, in the evaluation process, genes that were analyzed before 2019 but not after 2020 were removed from the result table.

#### Random Walk with Restart implementation

The Random Walk with Restart (RWR) algorithm was implemented using the PyRWR library (https://github.com/jinhongjung/pyrwr.git), a Python implementation. RWR is a graph-based algorithm that measures the proximity between a given seed node and all other nodes. It was applied to calculate node proximity within the STRING and FunCoup networks. All PyRWR parameters were used with default values. Cutoffs were implemented during the integration of RWR data into the LEXAS-plus model (STRING: 0.0005, FunCoup: 0.001).

#### Calculation of SHAP values

To interpret the importance of features in our XGBoost model, we calculated SHAP values using the TreeSHAP implementation in the SHAP Python library. This implementation is specifically designed for tree-based models like XGBoost.^56^ We created a TreeExplainer instance with the model, specifying 'tree_path_dependent' for feature perturbation. Then, we used this explainer to calculate SHAP values for our input data. The 'approximate=True' argument was passed to the shap_values method to speed up computation while maintaining a reasonable level of accuracy. The calculated SHAP values were then used to assess feature importance.

#### Experiment method prediction

Given the feature vectors used to train LEXAS model, a multi-class logistic regression classifier was trained to predict the category of the experiment method in the next experiment. Experiment methods were categorized into 20 groups as follows: knockdown/knockout, overexpression, immunofluorescence, protein-protein interaction, RT-PCR or qPCR, bioinformatics, immunohistochemistry, next generation sequencing, rescue experiment, protein structure, FISH, screening, mass spectrometry, super-resolution microscopy, electron microscopy, GWAS, live-imaging, Xray-flattering, circular dichroism and others. The accuracy of the method category prediction was 45.2%.

#### Web application

For the user interface, the LEXAS, LEXAS-plus and LEXAS-data models were trained using the information on all collected experiments and latest information sources as of 2023-01-30. When a user queries a gene, the top 100 genes with the highest probabilities are shown in the suggestion interface. The web application is built using Python, Flask, uWSGI and Nginx. The application is accessible through a web browser. To ensure data privacy and security, user inputs are encrypted using SSL/TLS protocols.

### Quantification and statistical analysis

Mann-Whitney U tests were conducted in Figures 3 and S2 using Python scipy library^57^ to calculate P values. P values were denoted as ∗∗∗ for P < 0.001 and NS for P > 0.05 (not significant). Bootstrapping tests were conducted in Figures 3 and S2 using Python to acquire 95% confidence interval. We performed 10,000 runs to calculate the mean values, using resampling with replacement.^58^

### Additional resources

LEXAS web interface: https://lexas.f.u-tokyo.ac.jp.

## Supplemental information

Document S1. Figures S1–S3 and Tables S1, S4, and S5

Table S2. The list of experiment method, related to Figure 2

Table S3. The number of experiments per gene, related to Figure 2

Table S6. Mean of AUROC@100 for genes classified by Gene Ontology terms, related to Figure 4
