# Supplementary material for: Predicting potential target genes in molecular biology experiments using machine learning and multifaceted data sources
Source: iScience. 2024 Feb 23;27(3):109309. doi: 10.1016/j.isci.2024.109309 (PMC10933549; doi:10.1016/j.isci.2024.109309)
Supplement: Document S1. Figures S1–S3 and Tables S1, S4, and S5 [file mmc1.pdf]

iScience, Volume *27*

Supplemental information

Predicting potential target genes in molecular biology experiments using machine learning and multifaceted data sources

Kei K. Ito, Yoshimasa Tsuruoka, and Daiju Kitagawa

Figure S1

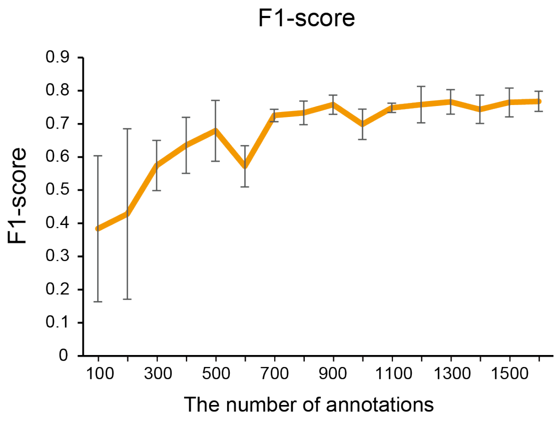

Figure S1. The relationship between annotation numbers and fine-tuned BioBERT performance, related to Figure 2

Line graph depicting the F1 score for the extraction of gene-experiment relationships according to the number of annotations. Data are presented as the mean +/- standard deviation.

Figure S2

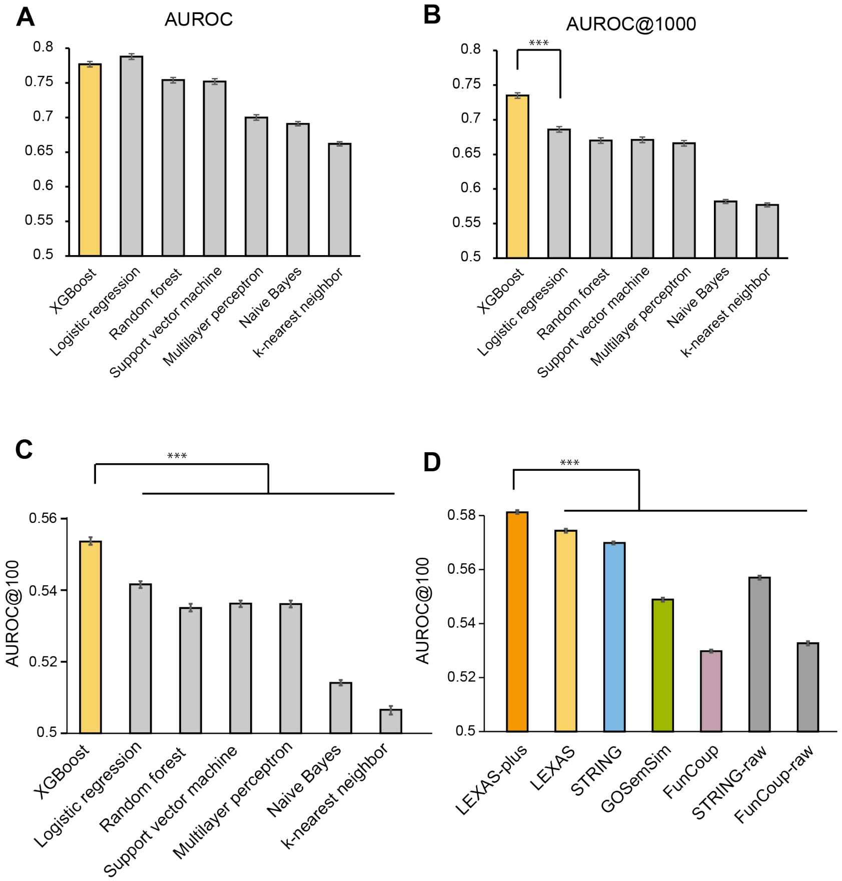

Figure S2. Evaluation of machine learning models, related to Figure 3

(A)(B) Comparison of prediction accuracy between algorithms. AUROC (a) and AUROC@1000 (b) were calculated using 7 different models. Data are presented as the mean AUROC or AUROC@1000 +/- 95% confidence interval (n=8278). **(C)** Comparison of prediction accuracy between algorithms where all genes examined after the query gene in the article were considered true positive. Data are presented as the mean AUROC@100 +/- 95% confidence interval (n=8278). **(D)** Comparison of prediction accuracy between our models and other resources where all genes examined after the query gene in the article were considered true positive. Data are presented as the mean AUROC@100 +/- 95% confidence interval (n=13381). Mann-Whitney U tests were used to compare the mean of AUROC and obtain the P values. ***, $P < 0.001$

Figure S3

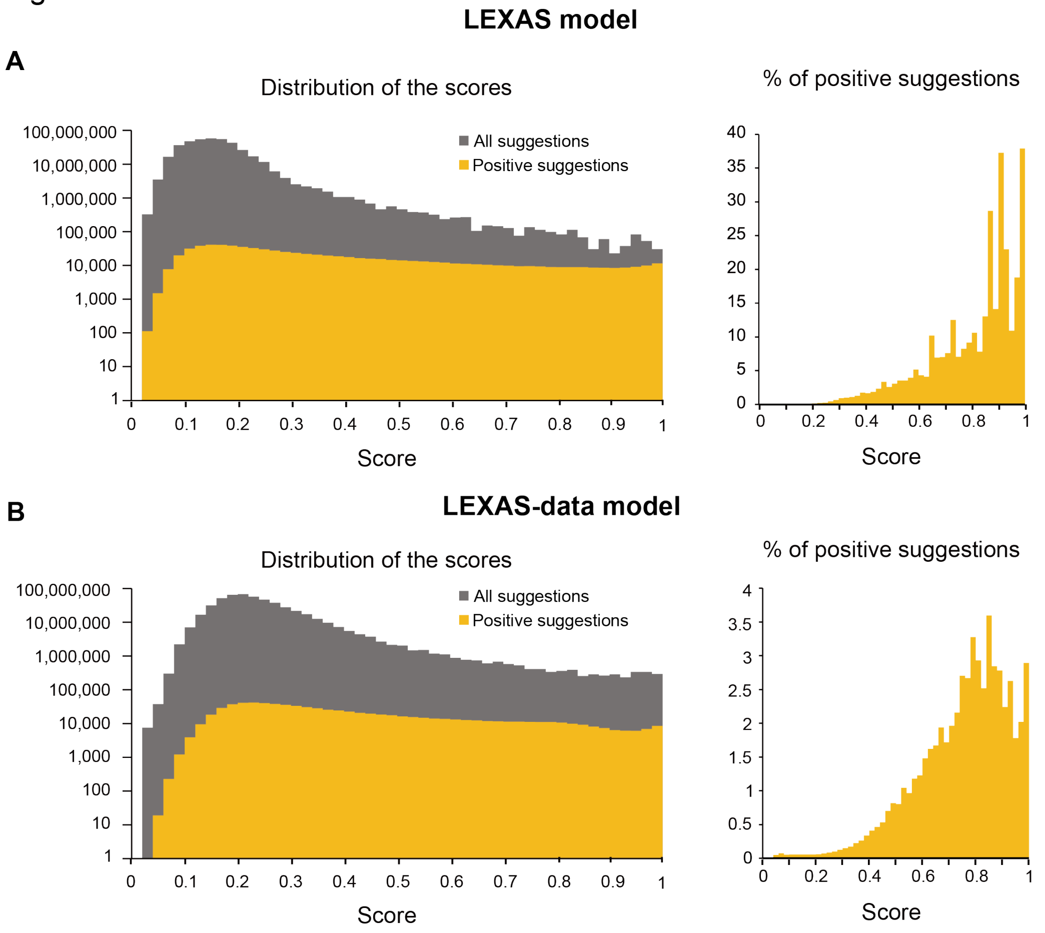

Figure S3. Analysis of scores provided by LEXAS and LEXAS-data, related to Figure 5

(A) The distribution of probabilities for all suggestions, positive suggestion, and the ratio of positive suggestions. The probabilities were calculated with the LEXAS model trained with the experiments from 2010 to 2018. **(B)** The distribution of probabilities for all suggestions, positive suggestion, and the ratio of positive suggestions. The probabilities were calculated with the LEXAS-data model trained with the experiments from 2010 to 2018. The bin width in the histograms is 0.02 in (A) and (B).

Table S1. The terms excluded from the original gene term list of HGNC, related to Figure 2

aid	dap	has	mts	set
albumin	dsp	hcc	name	sex
all	ecl	hek	nat	she
anova	ecm	his	not	sim
apo	end	hits	nude	simple
april	eng	ice	orf	sp2
aria	face	ids	osteosarcoma	sp5
bar	fact	impact	out	spatial
bars	fast	kit	pca	step
bca	fat	lab	per	task
best	fbs	large	pole	tbs
blast	fig	led	prism	tcs
bright	find	light	proc	tem
camp	fish	lps	red	thank
can	flash	mass	ref	tsa
cat	for	max	rest	tube
ccd	gap	men	ros	type
chip	gas	met	san	was
cis	get	mice	scs	yes
cut	goat	minor	sds	
damage	great	mri	sera	

Table S4. Mean of AUROC@100 among algorithms, related to Figure 3

Method	XGBoost	LR	RF	SVM	MLP	GNB	KNN
Just next	**0.581**	0.568	0.556	0.562	0.559	0.521	0.506
95%CI	0.578-0.584	0.565-0.571	0.553-0.559	0.559-0.565	0.556-0.562	0.519-0.522	0.505-0.507
All following	**0.553**	0.541	0.535	0.538	0.538	0.514	0.504
95%CI	0.551-0.555	0.540-0.543	0.534-0.537	0.537-0.540	0.536-0.539	0.513-0.515	0.504-0.505

Table S5. Mean of AUROC@100 among related tools, related to Figure 3 and Figure S2

Method	LEXAS-plus	LEXAS	STRING	GOSemSim	FunCoup	STRING-raw	FunCoup-raw
Just next	**0.573**	0.568	0.569	0.55	0.53	0.555	0.52
95%CI	0.571-0.575	0.567-0.570	0.567-0.571	0.548-0.552	0.529-0.532	0.553-0.558	0.517-0.533
All following	**0.581**	0.576	0.57	0.548	0.531	0.557	0.533
95%CI	0.580-0.582	0.574-0.578	0.568-0.571	0.546-0.550	0.530-0.532	0.554-0.560	0.531-0.535
